# Grandmother Spider: Connecting All Things

**Published:** 2006-12-15

**Authors:** Lynne S. Wilcox

The Spider Woman or Grandmother Spider legends are part of the creation mythology for several southwestern American Indian tribes, including the Hopi, Pueblo, and Navajo. One story says that in the beginning of time only two beings existed: Tawa, the Sun God, with the powers of all that is above; and Spider Woman, the Earth Goddess, with the powers of all that is below. The Sun God imagined the creatures of the earth, and Spider Woman turned these thoughts into living plants, animals, and people. She attached a thread of her spider silk to each person to provide access to her wisdom and protection (1).

The Centers for Disease Control and Prevention's (CDC's) Coordinating Center for Health Promotion (CoCHP) has a special focus on healthy people across the lifespan, one of the primary goals of CDC (2). During this year's National Health Promotion Conference (September 12–14, 2006), the partners of CoCHP came together to explore public health concerns in chronic disease, reproductive health, disability, birth defects, and genomics. CoCHP's mission requires an understanding of multiple topics, and each program within the coordinating center has depth and breadth in its body of scientific and programmatic knowledge. Yet all these fields address the same life stages across diverse populations; it is conceivable that a single individual might be a candidate for most of these programs at some point in life.

It is a paradox of public health that it must pull apart the elements of health to examine each aspect before putting them together again to implement successful programs. Like Spider Woman, professionals in these fields connect to a web of activities to promote good health. In this issue, *Preventing Chronic Disease* honors this web with reports that connect multiple concepts. We thank Dr. Karen Steinberg, senior science officer of CoCHP, for serving as guest editor for this issue. Start with any article and follow the threads. Find the thread that starts with physical activity and obesity concerns and connects to low-income women (3), American Indians (4), and Hispanic populations (5). Or the thread that travels through the life stages: children (6), young adults (7), midlife women (3), and older adults (8). Or other threads that identify connections among people, environments, and exposures of concern to public health practice.

More broadly, the public health web connects across diverse partners and programs. Here are a few examples within CoCHP:

- CDC's Healthy Aging Network unites nine university Prevention Research Centers (PRCs) to develop a research agenda that identifies the determinants of healthy aging and develops community-based programs to promote good health in older adults. The Cancer Prevention and Control Network brings PRCs together to focus on cancer prevention and control in community-based interventions. Other PRC networks include the Physical Activity Policy Research Network and the Cardiovascular Health Intervention Research and Translation Network (9).
- The Disability and Health program at CDC partners with the Spina Bifida Association, The National Center on Physical Activity and Disability, the Amputee Coalition of America, the Christopher and Dana Reeve Paralysis Resource Center, and the Special Olympics Healthy Athletes program to provide resources for people of all ages who have disabilities (10).
- CDC partners in the HuGENet, the Human Genome Epidemiology Network (11). This is a global collaboration for population-based genomic epidemiology. It includes research programs that obtain and contribute population data on human genes in the European Union, Australia, and North America.
- The Pregnancy Risk Assessment Monitoring System (PRAMS) is a partnership of CDC and state health departments to collect population-based data on women's behaviors before, during, and after pregnancy (12). It collects data on maternal behaviors such as smoking, vitamin use, and pregnancy planning for 38 states and New York City; this information is used to develop policy, design and evaluate state programs, and identify emerging issues in maternal and infant health.
- The CDC birth defects program supports the Centers for Birth Defects Research and Prevention, a network of universities and state health departments that participate in a joint birth defects research study and conduct additional birth defects research across the United States (13).

These examples imply additional layers of connections such as universities, professional organizations, and states that participate in multiple networks; populations that receive attention on multiple fronts; and health conditions that are addressed in multiple programs. The threads of these partnerships are numerous and overlapping.

The Spider Woman stories did not end with her role in the creation of the earth. She continued to protect her people by teaching them to grow corn, make clay pots, and spin and weave. In her grandmother role she provided advice as people traveled across the world to new homes, and through her silk threads she maintained the connections of all humankind. Like the web of connection that Spider Woman offered, public health requires an interconnected framework to support good health throughout our lives.
